# Trust undone: How COVID-19 coverage shaped scientists’ trust in journalism and their willingness to engage with the media

**DOI:** 10.1177/09636625261416830

**Published:** 2026-02-12

**Authors:** Frank Marcinkowski, Hella de Haas, Sarah Kohler

**Affiliations:** 1 Heinrich Heine University Düsseldorf, Germany; 2 Hochschule Düsseldorf University of Applied Sciences, Germany

**Keywords:** COVID-19 pandemic, media engagement, science communication, scientist-media relations, scientists’ trust in journalism

## Abstract

This study explores how scientists’ experiences during the pandemic influenced their trust in journalism and their willingness to engage with the media. The study employed a survey approach, collecting data from 4089 scientists affiliated with German universities and research institutions. Trust in journalism was measured across five dimensions: appropriate topic selection, accurate representation, proper fact selection, fair assessment, and desirable impact. Structural equation modeling was used to analyze the relationships between dissatisfaction with pandemic-era media coverage, trust in journalism, and scientists’ willingness to engage in science communication. Results show that scientists’ trust in news media is generally limited and varies across media types. The COVID-19 pandemic exacerbated distrust, particularly in media outlets expected to maintain high standards, such as national newspapers and public broadcasters. Trust in journalism proved central in mediating dissatisfaction and engagement, highlighting that distrust may reduce scientists’ media involvement and, in turn, weaken public trust in science.

## Introduction

Public trust in science has long been a widely debated issue in politics, the economy and the scientific community itself. The COVID-19 pandemic intensified these discussions, thrusting science communication into unprecedented relevance. As governments worldwide relied on rapidly evolving scientific knowledge to formulate pandemic responses, public compliance with policy measures became contingent not only on trust in political institutions but also on confidence in scientific expertise (Bicchieri et al., 2021; Intemann, 2023; Pagliaro et al., 2021). This interdependence between science and societal action rendered the pandemic a critical case study for understanding the dynamics of trust in science. Yet, the rise of anti-vaccine movements and the radicalization of opposition to public health measures, often framed as rejection of scientific consensus, have sparked concerns about a progressive erosion of trust in science and technology within Western democracies (Bromme et al., 2022; Franic, 2022). These developments have catalyzed a new surge of research examining public attitudes toward science (Agley, 2020; Cologna et al., 2025).

Paradoxically, while public trust in science has been scrutinized extensively, the reciprocal relationship, namely scientists’ trust in the news media and their audiences, remains underexplored. This gap is striking, given the pandemic’s dual role as both a public health crisis and a communication challenge. This study addresses this imbalance by investigating how scientists’ experiences during the pandemic shaped their trust in journalism and their perceptions of public rationality. We posit that repeated encounters with media misrepresentation, oversimplification of complex findings, or hostile public discourse may have eroded scientists’ confidence in the media’s ability to facilitate constructive dialogue. Such disillusionment could prompt researchers to withdraw from media engagement, despite institutional efforts to promote science outreach. This potential retreat carries significant implications. Scientists’ public engagement serves as a vital “access point” to abstract scientific systems, enabling “facework commitments” (Giddens, 1990: 83–92) that foster trust between experts and laypersons. When scientists participate in media interactions, they humanize institutional science, bridging the gap between specialized knowledge and public understanding. Conversely, withdrawal from public discourse risks severing these connections, diminishing opportunities for trust-building and reinforcing perceptions of science as an insular enterprise. A decline in scientists’ willingness to engage could thus initiate a feedback loop, further destabilizing public trust—a prospect with profound consequences for evidence-based policymaking and societal resilience in future crises. Hence, public trust in science and scientists’ trust in the public are two sides of the same coin.

Against this backdrop, our study makes three contributions to the literature on science communication and scientist–journalist relations. First, we advance an expectancy-based conceptualization of trust in news media, theorizing it as a multidimensional construct. Second, we operationalize this framework through a novel survey instrument, enabling empirical measurement of trust dimensions among scientists. Third, we analyze the interplay between pandemic-era experiences, media trust, and engagement intentions. Our analysis draws on data from a standardized survey of 4089 Web of Science (WoS)-indexed authors affiliated with German universities and research institutions. By focusing on scientists’ perspectives, this study illuminates the relationship between public trust in science and scientists’ trust in the public.

## State of the literature

In 1998, *Science* reported on a survey of 1400 US professional journalists and scientists. The findings revealed that only 2% of scientists expressed a high level of trust in television at that time, whereas 48% reported having no trust in it whatsoever. All others claimed to have some trust. Trust in the press was similarly limited: merely 11% of scientists indicated high trust, while 22% stated they had no trust at all (Mervis, 1998). Comparable, recent data of this kind are currently unavailable. In our literature review, we found only one other study that explicitly addresses the trust that scientists place in journalists. Drawing on 15 qualitative interviews with geneticists at Johns Hopkins University and 22 interviews with journalists who reported on their scientific findings, Geller and her coauthors underscore the central role of trust in enabling productive collaboration and high-quality science coverage (Geller et al., 2005). While the article does not offer a formal definition or conceptual analysis of trust, nor a corresponding measurement framework, trust is implicitly understood as the aggregate of conditions that, from the perspective of participants, characterize successful interactions between journalists and scientists. These conditions include ease of access, openness during conversations, a willingness to discuss sensitive or controversial topics, and ultimately, news coverage that satisfies both parties. The authors conclude that insofar as trust facilitates access for science journalists and enhances the quality of interviews, it may also contribute to improved media coverage.

The relationship between scientists and journalists has long been conceptualized as an encounter between distinct professional cultures, each governed by different norms, expectations, and operational logics (Peters, 1995, 2014). The majority of contributions do not address trust directly, but employ related constructs such as attitudes, perceptions, or evaluations. Employing surveys of 234 journalists and 448 scientific experts, Peters (1995) identified significant divergences in expectations and preferences concerning media interactions. Scientists valued accurate and precise representation of their findings, preferred pre-publication review, and emphasized the translation of jargon into accessible language. They also expected journalists to respect disciplinary boundaries and to promote the benefits of scientific research, though a somewhat paternalistic attitude toward media and audiences was evident. This worlds-apart approach was used in later studies to highlight similar gaps (Gunter et al., 1999; Maillé et al., 2009; Marín-González et al., 2023; Reed, 2001). Expanding this line, Peters and colleagues (Dunwoody et al., 2009; Peters et al., 2008) conducted an international survey of 1354 biomedical researchers in five countries. Scientists reiterated expectations for accurate reporting, avoidance of sensationalism, and respect for deadlines, reflecting a persistent concern for control over communication. Similar findings emerged in a qualitative study of neuroscientists in the United States and Germany (Allgaier et al., 2013). While acknowledging the benefits of media exposure, they remained cautious about journalistic practices, citing risks of sensationalism and misrepresentation.

To provide an updated account of scientists’ media relations across various disciplines and national contexts Peters synthesized findings from multiple survey studies conducted between 2005 and 2012 (Peters, 2013). His analysis confirmed that scientists widely perceive media visibility as important and consider responding to journalists a professional obligation. Media interactions are increasingly shaped by institutional norms and strategic considerations, with scientists emphasizing the need for pre-publication review to safeguard the accuracy of their representation. Extending this research to Brazil, Massarani and Peters (2016) found consistent expectations for accuracy, pre-publication consultation, and an educational role for science reporting. A perception of inaccuracies persisted. Galvão et al. (2021) confirmed these findings, showing that Brazilian scientists expect journalists to consult them prior to publication, maintain accuracy, and support public education.

Studies from Denmark (Møller Hartley, 2015) and the Netherlands (Dijkstra et al., 2015) reveal similar patterns. Scientists expect accuracy and sufficient journalistic understanding, and express frustration over oversimplification. MacGregor et al. (2020) review literature on co-production, showing researchers’ concern with accurate, non-sensationalist coverage and their recurring expectation of retaining some authority over narratives (see also Verkest, 2023). Negative perceptions often stem from past experiences of misrepresentation and loss of nuance.

COVID-19 renewed scrutiny of these relations. In Italy, Portugal, and Spain, most researchers engaged with media during the pandemic and rated experiences positively, motivated by the duty to inform and counter misinformation. Concerns nevertheless remained about sensationalism and lack of preparation. Practices that facilitated collaboration included provision of questions in advance and content review (Marín-González et al., 2023). De Jong et al. (2024) show that in the Netherlands, both scientists and journalists struggled with the complexity and societal impact of the pandemic. Researchers were ambivalent about whether their role should be purely informative or also persuasive, while journalists emphasized their role as independent gatekeepers.

Collectively, the literature illuminates a consistent pattern: scientists across different disciplines and cultural contexts value media engagement but harbor strong expectations regarding the fidelity, consultation practices, and educational function of journalistic reporting. Scientists express enduring concerns about the accuracy and control of science communication in the public arena. Where trust is addressed, it is treated as a necessary condition for interactions to take place, or as a favorable prerequisite for high-quality media reporting on scientific findings. According to our review, there is no standardized, comparable measure of scientists’ trust in journalism. Most studies rely on proxy variables, varying terminology (e.g. “attitudes,” “perceptions,” “confidence”), and methods (qualitative, quantitative) to approach the topic.

## Theory and hypotheses

### The concept of trust

In the tradition of sociological theorizing, we understand trust as a foundational mechanism for social cooperation, enabling relationships between individuals or groups who possess the capacity to act independently. Sociologists define trust as a dynamic in which one party voluntarily assumes risk by relying on another party to act in their interest, even when the trustor cannot directly control or observe the trustee’s behavior. This inherent uncertainty led Niklas Luhmann (1979: 24) to describe trust as a “risky investment” into the future, a metaphor emphasizing its forward-looking and vulnerable nature. Similarly, Mayer et al. (1995: 712) characterize trust as “the willingness to be vulnerable to the actions of another party.” For example, a patient undergoing cosmetic surgery trusts the surgeon to improve their appearance despite knowing the procedure could result in complications. The patient cannot oversee the surgeon’s actions during the operation, nor can they guarantee the outcome. This risk awareness is crucial; without it, the action might be perceived as naivety or recklessness rather than trust. From the trustee’s perspective, there is an understanding that their actions will be interpreted as either confirming or disappointing the trust placed in them (Barber, 1983; Lewis and Weigert, 1985). This awareness can influence their behavior, particularly in repeated interactions, though it does not diminish their autonomy in fulfilling their role (Seligman, 1997). Trust is rooted in the trustor’s expectations of the trustee’s future actions. These expectations are shaped by the trustee’s societal role, generating *normative* and *functional* standards. For instance, society expects surgeons to prioritize patient safety, a normative expectation tied to their professional duty, and journalists to report accurately, a functional expectation tied to their role in democratic discourse. Trust thrives in situations of managed uncertainty, where partial knowledge allows actors to navigate risk without full control. As Georg Simmel (1908[1999]: 393) observed, trust is the “intermediate state between knowledge and ignorance,” a social lubricant that enables collaboration even when outcomes cannot be guaranteed. In summary, trust is a complex and multifaceted concept that involves vulnerability, expectation, and the interplay between trustor and trustee.

### Scientists’ trust in journalism

In the following, we specify the standard elements of a trust relationship by applying them to the case of scientists’ trust in the news media, represented by individual journalists. Importantly, scientists are not considered here as consumers of news but as subjects of reporting or as journalistic sources. In this context, the act of trust consists of scientists delegating the dissemination of their knowledge and expertise to an independent body, namely journalism, rather than communicating it themselves via personal channels or avoiding public communication altogether. This delegation involves significant risks. Scientists are acutely aware of the possibility that their input might be inaccurately represented, that such misrepresentation could damage their own reputation or that of their institution, discipline, or the scientific enterprise more broadly. They also face the risk of being instrumentalized by seeing their statements used to advance agendas they do not endorse. More generally, they risk losing control over what is done with their knowledge once it enters the media sphere (Peters, 2013: 14107). Thus, by engaging with journalists, scientists expose themselves to vulnerability. If they proceed nonetheless, it is based on the expectation that journalistic coverage will at least not be harmful and may even be beneficial to themselves, to their work, or to public understanding. Journalists, in turn, are aware that they are being trusted. One of the core tenets of professional journalism recognized globally is to treat sources with care and credibility (Hanitzsch et al., 2019). This mutual awareness frames the trust relationship.

This leads to the question of which expectations these are in detail. Based on relevant work on audiences’ trust in news media (Kohring and Matthes, 2007) and the literature on relationships between scientists and journalists referred to above, we assume that five expectations constitute the core dimensions of the trust relationship under consideration. First, scientists generally expect that interviews will focus on topics within their own area of demonstrated expertise. For example, a biologist would not typically be expected to comment on astrophysics, nor would a sociologist of religion be asked to evaluate voter behavior. We term this the “appropriate-topic-selection expectation.” It reflects academic standards of legitimacy and peer recognition, since public commentary outside one’s field may be viewed unfavorably by fellow academics (Ivanova et al., 2013; Peters, 2013: 14106; Peters et al., 2008; Rödder, 2012).

Second, scientists expect that the knowledge they provide will be accurately represented in the resulting media coverage (Gunter et al., 1999; Marín-Gonzalez et al., 2023; Massarani and Peters, 2016; Reed, 2001). This expectation is grounded in the normative framework of professional journalism, where truthfulness, factual accuracy, and objectivity are regarded as foundational principles (Hanitzsch et al., 2011). Scientists, whose own professional ethos is likewise centered on the pursuit of accuracy and truth, are particularly sensitive to the faithful reproduction of their statements in public discourse. They expect that the information they provide will not be misrepresented or taken out of context in ways that fundamentally alter its meaning.

Third, scientists expect that journalists will select content appropriately from the information provided. This can be termed the “expectation of appropriate fact selection,” and it is closely linked to the previously discussed expectation of accurate representation. This does not mean they anticipate comprehensive coverage. Journalism must simplify and select due to constraints of space, time, and accessibility. However, scientists expect that the selection process will privilege the most salient or scientifically relevant aspects of their input (Geller et al., 2005; Verkest, 2023). Trust is undermined when this selection appears skewed, for example, when sensational details are emphasized at the expense of substance, or when risk is highlighted without contextualizing benefits (Gunter et al., 1999).

A fourth expectation concerns the evaluative framing of the scientist’s work. Media users are aware that journalism does not merely convey factual information but also interprets, contextualizes, and comments on it (e.g. Kohring and Matthes, 2007 ). Consequently, scientists expect that the journalistic assessment of their research or of the broader state of knowledge in their field will be both informed and proportionate in terms of its scientific and societal relevance (Dijkstra et al., 2015; Besley and Nisbet, 2011). This “expectation of appropriate evaluation” can be violated in two principal ways: either through unjustified amplification, often referred to as *scientific hype* (Allgaier et al., 2013; Dijkstra et al., 2015; Intemann, 2022; Marín-Gonzalez et al., 2023; Weingart, 2017), or through the neglect of research findings that the scientist considers particularly significant. Such misalignments can lead to dissatisfaction, as scientists generally anticipate a level of interpretive competence that reflects journalistic responsibility and epistemic sensitivity (Peters, 2013).

Finally, scientists often harbor expectations regarding the impact of media coverage on public understanding (Allgaier et al., 2013; Dunwoody et al., 2009; Peters et al., 2008; Tsfati et al., 2010). We refer to this as the “journalistic impact expectation.” This expectation reflects the underlying assumption that science-based reporting contributes to an increase in the audience’s knowledge, awareness, or critical understanding of scientific issues. While this expectation is normatively significant, it remains empirically difficult to verify. Scientists may look to public discourse, policy developments, or behavioral changes in specific social groups as indirect measures of journalistic impact. We conceptualize this impact expectation as integrating a specific form of *trust in the public* into the broader, multidimensional construct of *trust in journalism* developed in this study. It reflects not only confidence in journalistic professionalism, but also an implicit belief in the public’s capacity to engage meaningfully with scientific content when it is appropriately mediated.

The five expectations discussed should not be seen as exhaustive. Scientists may also value interpersonal qualities such as respect, responsiveness, and communicative clarity. Our aim, however, is to identify the most central and generalizable expectations that define the trust relationship. The delimitation of our model is thus guided by the principle of parsimony. Drawing on established models of media trust (e.g. Kohring and Matthes, 2007; Tsfati and Cappella, 2003), we argue that these core dimensions are both conceptually robust and empirically measurable.

How can scientists assume that their trust will not be betrayed, that they will not be “hurt” in a given context, even though they make themselves vulnerable? We assume that expectations of interactions with news media are initially fed by firsthand experience. Every researcher who has ever made himself or herself available as a news source for journalistic products has had experience of this. As a rule, they will extrapolate these experiences and assume that it will be the same the next time. Each new experience expands the basis for the projection. Furthermore, it can be assumed that academics share their experiences with the news media among each other. This provides additional access to secondhand experiences that colleagues or peers have had and report on in conversations or otherwise. Ultimately, it can be assumed that scientists form expectations of successful interaction between science and journalism on the basis of their news consumption. The more intensively someone receives journalistic products with scientific content in newspapers, television and radio, the more precise their idea will be of what to expect. During the COVID-19 pandemic, the news media was an important source of information. It can be assumed that scientists as news consumers were particularly interested in the media portrayal of the role of scientific research as a whole and the way in which the particularly visible protagonists of anti-COVID research were depicted by the media. Over a period of more than 2 years, every attentive media user was able to gather a multitude of impressions of the media’s dealings with science and scientists. The sheer length and intensity of these experiences is likely to have had a lasting impact on scientists’ expectations of the media. We therefore assume that the more critical a scientific observer’s assessment of the media’s performance during the COVID-19 crisis is, the lower their expectations of the media’s capabilities and, consequently, their trust in the news media. The second assumption follows directly from the theoretical framework outlined above. According to this, trust is the basis of every risky investment, such as the contact of a scientist with the news media. In other words, we hypothesize a positive correlation between trust in the news media and the personal willingness to engage with them for an interview or background discussion. From both assumptions together, it follows implicitly that critical assessments of the performance of news media during the pandemic have a negative indirect effect on the willingness of individual scientists to engage in mediated science communication.

In the following, we confront these assumptions with data from a survey of scientists that was conducted at the same time as the World Health Organization (WHO) announced the end of the pandemic in May 2023.

## Sampling and data collection

We sampled scientists with at least one peer-reviewed scientific publication in the 3 years preceding our data collection (2019–2022). Similar to other researchers (Allum et al., 2023; Economist Impact, 2022), we used WoS as our database to collect email addresses provided on scientific articles. After collecting articles, we checked if the author was affiliated with a German university or research institution, and checked for duplicates, TLDs (.de, .com), and syntax errors. In all, 53,718 randomly selected email addresses were contacted over the course of five days with two reminders from 11 April to 20 May 2023. With 14.9% failed deliveries due to, for example, change of workplace, 45,703 scientists (85.1%) received our survey invitation. In all, 4135 scientists completed the questionnaire, giving a response rate of 9.0%, which is comparable to that in other scientist surveys (Besley et al., 2018). After removing incomplete or inconsistent cases, a final sample of *N* = 4089 scientists remained for our analysis. Detailed information about the composition of the sample can be found in Table 1.

**Table 1.** Sample characteristics: percentages for gender, position, field of science, and affiliation (*N* = 4089).

Variables	%
Gender	
Male Female Other Missing	60.9 34.3 0.4 4.4
Position	
Doctoral student Post-doc Professor Researcher Other Missing	16.8 23.1 17.8 25.6 16.6 0.1
Field	
Humanities & Social sciences Life sciences Natural sciences Engineering & technology Interdisciplinary Missing	21.2 36.4 18.0 16.5 1.5 6.5
Affiliation	
Research university University of applied sciences Research institutes University clinic Research department in a company Research department of public administration Other Missing	40.1 5.1 23.1 9.7 3.7 4.7 10.7 3.0

## Measures

### Trust

Based on the theoretical considerations outlined above, 20 items were formulated, four for each of the five trust dimensions. By agreeing or disagreeing with these statements, the respondents were asked to indicate what expectations they have when they are contacted for an interview or background conversation by a news medium. Item formulations were evaluated in several rounds of discussion with experienced colleagues and adapted where necessary. The complete questionnaire, including the trust measurement, was subjected to a pre-test in March 2023, in which *N* = 416 randomly selected scientists from the original sampling frame took part. The evaluation of the test data set and the open feedback from the respondents led to various adjustments to the questionnaire. Among other changes, three trust items were deleted, resulting in a number of 17 trust items for the final survey. The wording of some items was finally modified. Although the trust measurement had generally worked quite well in the test, one crucial change was made to the survey design: Several respondents reported that they could not answer the question without first specifying the type of media. We therefore decided to ask the questions of trust in the context of five different scenarios. All scenarios were introduced with the identical wording: “Imagine you are approached by . . . (media type) . . . who is interested in your scientific expertise on a current topic. What expectations do you have for the conversation?” “Public broadcaster (radio or television),” “commercial broadcaster (television or radio),” “local daily newspapers,” “national daily newspapers” and “tabloid newspapers” were the subject of the five scenarios. All respondents were randomly assigned to one of the five settings. Following the introduction, respondents were then asked to indicate how confident they were in their own expectations: “Based on what you have experienced or heard, to what extent are you confident that the journalist . . . will ask you about a topic that you have been researching?” (*appropriate choice of topic).* Further examples of item wording read “. . . will correctly report the information you have provided?” (*accurate reproduction of scientific knowledge*), “. . . will properly explain to the audience the relevance of scientific expertise on the topic?” (*appropriate assessment*), “. . . will concentrate his reporting on the scientific facts that really matter?” (*appropriate selection of facts*), “. . . will improve the audience’s understanding of the scientific foundations of the topic?” *(journalistic impact)*. Answers were coded on a 5-point scale, from 1 = “not at all confident” to 5 = “very confident.”^1^

To test the adequacy of the measurement we conducted a confirmatory factor analysis (CFA), whereby in the first step a measurement model with five correlated latent variables is implied. Compared to the most frequently cited standard for good model fit (Hu and Bentler, 1999), all indices reflect a good match between the model and the data: chi-square 1707.2834 (109), *p* < .001, root mean square error of approximation (RMSEA) = .0606 [.0581, .0631], comparative fit index (CFI) = .9661, standardized root mean square residual (SRMR) = .0355. All original items exhibit a factor loading of at least λ = .74. The average extracted variance ranged between 58% (Fc1 = choice of topic) and 75% (Fc5 = impact expectation). A multi-group CFA was conducted to examine the measurement invariance of the CFA across the different media scenarios. All models are statistically significant, and the chi-square difference tests between the unrestricted model and the increasingly restricted (or “smaller”) models also yield significant results (see Table A2 in the Supplemental Material). Strictly speaking, this suggests that the measurement model does not function equally well across the different respondent groups exposed to varying media scenarios. However, given the complexity of the measurement model, comprising 17 observed and 5 latent variables, and the large sample size (*N* > 4000), to which the chi-square test is particularly sensitive, it is advisable to rely on alternative fit indices for model comparison (Putnick and Bornstein, 2016). Of particular interest is the assumption of strict or scalar invariance, as it permits direct and meaningful comparisons of latent mean scores across groups. In this context, the differences in CFI, RMSEA, and SRMR between the unrestricted model and the model with scalar invariance remain well below the recommended thresholds of ΔCFI ⩽ −.01, ΔRMSEA ⩽ .015, and ΔSRMR ⩽ .030 (Chen, 2007; Rutkowski and Svetina, 2014). We conclude that scalar invariance of the measurements can be assumed in the various media scenarios.

The discriminant validity between the five factors was checked with recourse to the Fornell–Larcker criterion (Fornell and Larcker, 1981: 41), according to which a latent construct to be estimated should on average share a higher proportion of variance with the respective indicators (average variance extracted (AVE)) than with any other latent construct within the model. Nine of the ten pairwise comparisons satisfy the criterion. However, the squared correlation of the factors *accurate reproduction of knowledge* and *selection of facts* (*r*^2^ = .7955) exceeds the average extracted variance of both factors (AVE Fc3 = .6843; AVE Fc4 = .6707). Thus, even though the dimensions can be conceptually separated as shown above, it cannot be assumed that the respondents were able to reproduce this difference on the basis of the items available to them.

**Figure 1.**
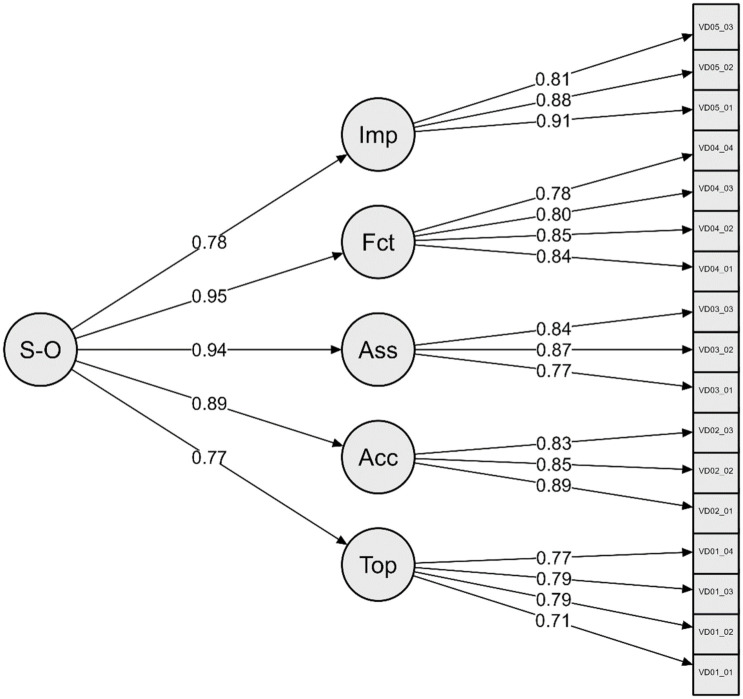
FIML CFA for second-order-factor model of trust in news media with standardized parameters.

Following our theoretical intuition, in the next step, we look for a common factor behind the original five dimensions. This means that the five latent constructs are modeled as indicators of a higher-order factor, which represents scientists’ trust in the news media. Figure 1 shows the measurement model with standardized path coefficients. As can be concluded from the global model fit indices, the second-order factor model fits the data just as well as the one initially tested: chi-square 1781.0206 (114), *p* < .001, RMSEA = .0605 [.0580, .0630], CFI = .9647, SRMR = .0367. According to Chen (2007) and Cheung and Rensvold (2002), a CFI difference of 0.0014 implies that the model fit of the second-order factor model does not deteriorate considerably. So there is no reason not to continue working with this smaller model.

The standardized factor loadings of the five latent constructs are at least λ = 0.77 or higher, which means they are very good indicators of the higher-order factor. The average extracted variance of all constructs is above the target value of 50% (Topic AVE = .5823; Accuracy AVE = .7300; Assessment AVE = .6844; Selection of facts AVE = .6709; Impact AVE = .7512). A closer inspection of the factor loadings indicates that expectations related to the selection of facts and journalistic judgment account for the largest share of variance in trust, followed by expectations concerning accurate representation. Expectations related to impact and topic selection exert comparatively weaker effects on scientists’ trust in journalists. Measurement invariance of the higher order factor model was validated using the multigroup CFA. The strong empirical fit of the scalar invariance model to the data (chi-square 2653.975 (678), *p* < .001, RMSEA = .060 [.058, .063], CFI = .953, SRMR = .051) suggests that the measurement operates consistently across different conditions, thereby supporting the modeling of trust as a higher-order factor (see Table A3 in the Supplemental Material).

### Independent variable

To measure scientists’ assessment of news media performance during the pandemic, we used two items covering both the quality of reporting and journalism’s treatment of scholars. The item wording was as follows: “The media coverage of the role of science during the COVID19 pandemic did not meet my standards” and “I did not like the way journalists treated some scientists during the pandemic.” Answers were given on a 5-point scale, from 1 = “strongly disagree” to 5 = “strongly agree.”

### Dependent variable

As a dependent variable, we measured the general willingness to appear in the news media and the specific intention to do so in the foreseeable future. The question about readiness was posed as the initial question of the survey and read: “How willing are you currently as a scientist to talk to the news media?” Answers were given on five levels from “1 = not at all willing” to “5 = very willing.” At the end of the questionnaire, we asked the scientists about their behavior toward the news media in the foreseeable future and whether they agreed or disagreed with the following statement: “I will express my views in the mass media when I have the opportunity to do so.” Here, too, the answers were given in five levels from “1 = very unlikely” to “5 = very likely.”

We assume that the two observed independent and dependent variables each represent a common factor, which we refer to as “discontent with the performance of the news media” during the pandemic and “willingness to science communication via news media.” Since measurement models for latent factors with only two observed variables are not testable on their own, we estimate both models together as a correlated factor model. The global fit indices reveal that the measurement model fits the data quite well: Chi-Square 0.0132 (1), *p* = 0.9084, RMSEA = .0000 [.0000, .0172], CFI = 1.0, SRMR = .0003. The AVE for the first factor (0.4010) is below the recommended threshold (0.50) but satisfactory for the second factor (0.5806). The mean value for willingness is *M* = 2.859 (*SD* = 1.167), which is just below the neutral midpoint of the 5-point scale and indicates a limited readiness among the scientists surveyed to engage in mediated science communication. The mean value of the independent variable dissatisfaction with the media is *M* = 3.489 (*SD* = 0.877), indicating widespread discontent with the media among the surveyed population. The bivariate correlation between the independent and dependent variables is weak but statistically significant (*r* = .18).

## Results

Before we turn to the test of the hypotheses, it is worth taking a look at the descriptive results of the trust measurement. Since we previously established strict equivalence of the measurement models between the groups, we can interpret these differences in a meaningful way. To this end, Table 2 shows the mean values of the five trust dimensions described above for each individual media genre. Overall, scientists’ trust in the news media is fairly limited. Only a few mean values are (slightly) above the neutral mean of the 5-point scale, most are more or less clearly below it. No means approached the upper end of the scale. Respondents have the least confidence in the correct selection of scientific facts reported in the news media. Trust in the correct reproduction of scientific evidence is also comparatively low. In contrast, journalists’ choice of topics received the highest level of trust. A comparison of media genres shows that tabloid newspapers are the least trusted when it comes to reporting scientific findings. The mean values of this group are at least half a scale point behind the comparison groups on all five dimensions.

**Table 2.** Means and standard deviation for five trust dimensions by media genres.

Trust dimension	Tabloid newspaper	Local newspaper	National newspaper	Public television	Commercial television	Overall
Choice of topic	2.4252^a,b,c,d^ (.86493) *N* = 658	3.1252^a^ (.87684) *N* = 587	3.2385^b^ (.89817) *N* = 587	3.2279^c^ (.87946) *N* = 577	3.1328^d^ (.82928) *N* = 704	3.0192 (.92188) *N* = 3113
Accurate reproduction	1.8837^a,b,c,d^ (.83381) *N* = 801	2.6582^a,b^ (.95023) *N* = 681	2.8285^b^ (.99719) *N* = 696	2.7000^c^ (.94066) *N* = 681	2.6541^b,d^ (.88992) *N* = 822	2.5279 (.98266) *N* = 3719
Selection of facts	1.7163^a,b,c,d^ (.72456) *N* = 800	2.3663^a,b^ (.82073) *N* = 705	2.5352^b^ (.85639) *N* = 689	2.4023^b,c^ (.82869) *N* = 663	2.3775^b,d^ (.77547) *N* = 806	2.2651 (.85197) *N* = 3663
Appropriate assessment	1.9590^a,b,c,d^ (.79985) *N* = 772	2.6510^a,b^ (.84660) *N* = 637	2.8458^b^ (.87336) *N* = 657	2.7213^c^ (.88215) *N* = 622	2.6787^b,d^ (.82305) *N* = 746	2.5514 (.90338) *N* = 3434
Impact	2.2332^a,b,c,d^ (.94987) *N* = 822	2.9633^a^ (.87559) *N* = 717	3.0730^b^ (.87040) *N* = 712	2.9748^c^ (.88649) *N* = 689	2.9976^d^ (.83057) *N* = 835	2.8347 (.93930) *N* = 3775

a,b,c,d Mean values with identical letters differ significantly at *p* < .05 according to post hoc statistics.

According to the Games–Howell post hoc analysis (Games and Howell, 1976), all mean differences between tabloid media and four other media genres are statistically significant. Thus, expectations for the proper functioning of the tabloid media are quite low, especially with regard to the accuracy of their reporting. In contrast, national newspapers enjoy the highest level of trust, ahead of public broadcasters. However, the mean differences between national newspapers and public broadcasting are statistically significant in just one dimension (selection of facts). Overall, three groups of media emerge when it comes to scientists’ trust in news media: tabloid media are by far the least trusted by scientists, while national newspapers and public broadcasters enjoy a significantly higher level of trust. In between, local newspapers and private television display comparable levels of trust.

**Figure 2.**
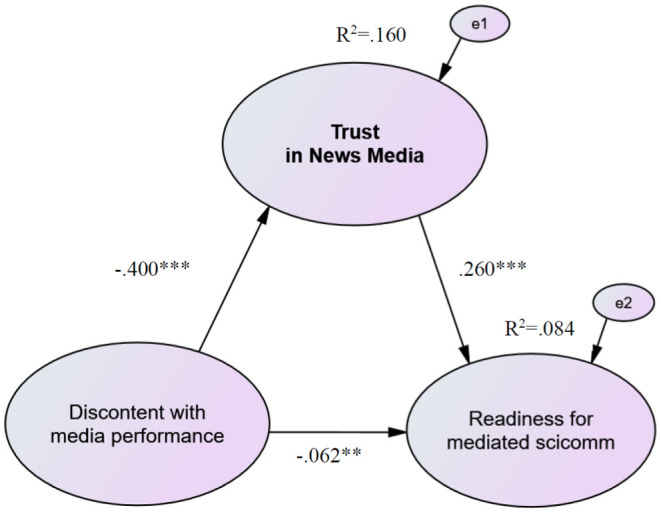
Structural model of causal relations among discontent with the performance of news media during the pandemic, trust in news media, and individual readiness for science communication via news media with standardized estimates. *N* = 4.089, ***p < .001; **p < .050.

Figure 2 presents the test of hypotheses by using structural equation modeling with full information maximum-likelihood estimation and standardized path coefficients. The specified model provides a close fit to the data as indicated by the global fit indicators: chi-square 2002.066 (181), *p* < .001, CFI = 0.964, RMSEA = 0.050 [.048, .052], SRMR = 0.034. As hypothesized, dissatisfaction with the media coverage of science and scientists during the pandemic has a medium negative effect on the generalized trust of the respondents in news media. The predictor alone explains 16% of the variance in scientists’ trust. The assumption that trust in the news media is an important prerequisite for scientists’ willingness to serve as a news source for science information is also confirmed. The correlation is moderate (*r* = .26) and explains about 8% of the variance of the dependent variable.^2^

The analysis reveals a significant negative indirect effect of scientists’ experiences with the press during the pandemic on their willingness to engage in science communication via the news media (*B* = –.138, *SE* = .017, *p* < .001, ß = –.104). While the direct association between the independent and dependent variables is fairly weak (*r* = −.06), it remains statistically significant. The results suggest partial mediation: trust in journalism partially transmits the effect of prior media experiences on the willingness to communicate via news outlets. The total effect amounts to *B* = –.221, *SE* = .036, *p* < .001, ß = –.166.

Finally, we tested the structural equation model separately for each media type. The fit measures obtained for multigroup structural equation modeling (SEM) were as follows: chi-square: 3096.159 (984), *p* < .001, RMSEA = .051 [.049, .053], CFI = .954, SRMR = .052. Table 3 shows the regression coefficients for the three paths of each model, including standard error and statistical significance.

**Table 3.** Path coefficients and standard errors for multigroup SEM.

Group	Predictor		Dependent	*B*	*SE*	*p*	ß
Tabloid Newspaper	Discontent	➝	Trust in news media	−0.178	0.034	<.0001	−0.261
	Trust in news media	➝	Readiness	0.217	0.081	<.0001	0.119
	Discontent	➝	Readiness	−.236	0.070	<.0001	-.190
Local Newspaper	Discontent	➝	Trust in news media	−0.293	0.041	<.0001	-0.421
	Trust in news media	➝	Readiness	0.645	0.097	<.0001	0.355
	Discontent	➝	Readiness			n.s.	
National Newspaper	Discontent	➝	Trust in news media	−0.375	0.044	<.0001	-0.518
	Trust in news media	➝	Readiness	0.598	0.105	<.0001	0.330
	Discontent	➝	Readiness			n.s.	
Public TV	Discontent	➝	Trust in news media	−0.314	0.040	<.0001	-0.457
	Trust in news media	➝	Readiness	0.553	0.100	<.0001	0.300
	Discontent	➝	Readiness			n.s.	
Commercial TV	Discontent	➝	Trust in news media	−0.322	0.041	<.0001	−0.475
	Trust in news media	➝	Readiness	0.780	0.108	<.0001	0.380
	Discontent	➝	Readiness			n.s.	

The analysis confirms that the general structural relationships of the model are consistently observed across the various media types examined. Specifically, higher levels of dissatisfaction with the performance of news media during the COVID-19 pandemic are associated with lower levels of scientists’ trust in journalism. Concurrently, trust in journalism emerges as a significant precondition for scientists’ willingness to engage in media-based science communication. However, the direct path between independent and dependent variables is only significant in the tabloid media model. In all other cases, we are dealing with a complete mediation of the relationship through trust in journalism. Furthermore, the explanatory power of the predictors differs markedly across media outlets. In the case of national newspapers, dissatisfaction with reporting exerts a substantially stronger negative influence on trust compared to tabloid newspapers. Conversely, trust in tabloid media proves to be a notably weaker predictor of scientists’ readiness to act as sources for journalistic reporting than in the case of all other media outlets: less than 2% of willingness in the tabloid media model, but nearly 13% in the context of local newspapers.

## Discussion

This article theorizes scientist–journalist relations by introducing scientists’ trust in journalists as a mediating mechanism that shapes the quality, frequency, and outcomes of their interactions. We build on an expectancy-based understanding of trust, according to which trust entails future-oriented expectations that enable individuals to engage in social interactions in which they render themselves vulnerable. In the context of interactions with journalists, we argue that scientists expect appropriate topic selection, accurate science reporting, proper fact selection, fair assessment, and enlightening impact. Empirical work employing the five-dimensional trust framework provides preliminary support for the validity of this conceptualization. In our sample, however, fact selection and accuracy of presentation were interpreted as synonymous. Consequently, the measurement model needs to be further improved in this respect. All dimensions proved to be statistically strong indicators of the latent construct, with expectations regarding journalistic judgment, fact selection, and accuracy of presentation slightly outweighing those concerning topic selection and journalistic impact. The suitability of the impact dimension is particularly noteworthy as it demonstrates that trust in journalism also encompasses expectations of audience reception, a factor that has received little scholarly attention to date. Overall, our multidimensional trust framework offers a nuanced analytical tool for future research, addressing an area that the literature on scientist–journalist interactions has hitherto neglected. From a practical standpoint, the model delineates the expectations of academic sources that journalists should respect in order to safeguard those sources’ trust.

Three empirical findings are particularly worth of discussion. First, scientists’ trust in news media is generally low. Given that many voices in the literature consider trust to be the single most important prerequisite for fruitful interactions between scientists and journalists, this is a worrying finding. Unfortunately, our cross-sectional data do not allow us to determine the extent to which the findings represent only a snapshot under the immediate influence of the pandemic. However, trust varies across media types. Rather than simply trusting or distrusting the media as a whole, scientists make subtle distinctions. The study reveals marked differences, with national newspapers and public broadcasters exhibiting comparatively higher levels of trust than tabloid newspapers. This likely reflects perceptions of professionalism, editorial standards, and historical accuracy in the former, whereas the reputation of tabloids as sensationalist outlets appears to amplify scientists’ fear of misrepresentation.

Second, scientists’ trust in the media is negatively associated with their experiences during the COVID-19 pandemic. Dissatisfaction with pandemic-era media coverage which was characterized by politicization, oversimplification, and distortion of scientific findings has eroded trust. Differences across media types can again be explained through the expectancy-based approach: where expectations of media performance are already low, as in the case of tabloid outlets, disappointments have a comparatively minor effect. In contrast, perceived failures of national newspapers and public broadcasters, where expectations are high, exert a stronger negative impact on trust. We may assume, pending further empirical confirmation, that this mechanism extends beyond the pandemic context. If so, news media contribute continuously, through their science reporting, to either strengthening or eroding the trust of potential academic sources.

Third, our results show that trust in journalists is a significant predictor of scientists’ willingness to act as news sources. Trust mediates, in particular, the effect of past media experiences on such willingness. Many factors influence scientists’ decisions to engage publicly, and trust can only account for a limited share of the variance in communication behavior. It nonetheless emerges as a decisive factor: all else being equal, scientists with higher levels of trust in journalism are more inclined to make their expertise available for media reporting.

Taken together, these three findings suggest that, among many other consequences, the pandemic has also reduced scientists’ willingness to participate in mediated science communication because their trust in journalism and its audience has suffered. As noted at the outset, the broader societal implications of this trend are considerable. A withdrawal of scientists from public discourse risks initiating a self-perpetuating cycle of disengagement, in which the absence of expert contributions deprives society of authoritative knowledge, thereby amplifying misinformation and eroding public trust in science. Such a development is particularly critical in the context of future crises that demand high levels of scientific literacy.

Like all scientific research, this study has its limitations. The first one concerns the geographic and cultural scope of the study. The analysis is based exclusively on scientists working in Germany, which restricts the extent to which the findings can be generalized to other national or cultural contexts. Media systems, political environments, and public trust in journalism vary considerably across countries. For example, in parts of Europe, trust in public broadcasters tends to be relatively high, whereas in other regions media trust levels may be lower or shaped by different historical and institutional factors. These contextual differences may influence scientists’ attitudes toward media engagement and therefore limit the applicability of the results beyond the German setting. A second limitation relates to the reliance on self-reported data. Participants’ willingness to engage with the media was measured through their own accounts, which may be influenced by social desirability bias, selective memory, or personal interpretation of the questions. Self-reported intentions do not necessarily correspond to actual behavior. Incorporating objective indicators, such as verified records of interviews, opinion pieces, or other documented media appearances, would provide a more accurate and comprehensive understanding of scientists’ engagement practices. A third limitation is the cross-sectional design of the study, which makes it impossible to establish causal relationships between the variables examined. While the findings suggest associations between trust in media and willingness to engage, they cannot statistically determine whether changes in trust lead to changes in engagement or vice versa. Longitudinal research that follows participants over time, particularly before and after significant events such as public health crises, would allow for a more precise assessment of causal pathways and the durability of observed patterns.

## Supplemental Material

sj-docx-1-pus-10.1177_09636625261416830 – Supplemental material for Trust undone: How COVID-19 coverage shaped scientists’ trust in journalism and their willingness to engage with the media Supplemental material, sj-docx-1-pus-10.1177_09636625261416830 for Trust undone: How COVID-19 coverage shaped scientists’ trust in journalism and their willingness to engage with the media by Frank Marcinkowski, Hella de Haas and Sarah Kohler in Public Understanding of Science

## Notes

1. The complete wording of the questions for all variables in the analysis can be found in the supplemental material.

2. For purposes of robustness assessment, we re-estimated the model by substituting the latent dependent construct with its two observed indicators (general willingness and concrete intention). The standardized regression coefficient of trust in journalism on general willingness amounts to β = .19 (*p* < .001), whereas its standardized association with the intention to contact the media is β = .21 (*p* < .001). Fit indices remain essentially unchanged across both alternative specifications. These results provide additional evidence for the stability of the reported conclusions.
