# Supplementary material for: Trust undone: How COVID-19 coverage shaped scientists’ trust in journalism and their willingness to engage with the media
Source: Public Underst Sci. 2026 Feb 12;35(4):490–507. doi: 10.1177/09636625261416830 (PMC13096614; doi:10.1177/09636625261416830)
Supplement: sj-docx-1-pus-10.1177_09636625261416830 – Supplemental material for Trust undone: How COVID-19 coverage shaped scientists’ trust in journalism and their willingness to engage with the media [file sj-docx-1-pus-10.1177_09636625261416830.docx]

**Trust Undone: How COVID-19 Coverage Shaped Scientists’ Trust in Journalism and their Willingness to Media Engagement**

**Supplemental Material**

Frank Marcinkowski^1^*,* Hella de Haas^2^*,* Sarah Kohler^1^

^1^ Heinrich Heine University Düsseldorf, Department of Social Sciences

^2^ Hochschule Düsseldorf University of Applied Sciences

**Contents**

Table A1: Item wording of variables used in the analysis

Table A2: Test of measurement invariance for CFA with correlated factors

Table A3: Test of measurement invariance for CFA with second order factor

Table A1: Item wording of variables used in the analysis

| Variables | Type | Items | Levels in analyses |
| --- | --- | --- | --- |
| discontent | independent variable | The media coverage of the role of science during the COVID‑19 pandemic did not meet my standards  [Die Medienberichterstattung über die Rolle der Wissenschaft in der Corona-Pandemie entsprach nicht meinen Ansprüchen] | 1 = stongly disagree, 5 = stongly agree  [1 = stimme überhaupt nicht zu, 5 = stimme voll und ganz zu] |
| discontent | independent variable | I did not like the way journalists treated some scientists during the pandemic  [Ich fand es nicht richtig, wie die Journalistinnen und Journalisten während der Pandemie mit einigen Wissenschaftlerinnen und Wissenschaftlern umgegangen sind] | 1 = stongly disagree, 5 = stongly agree  [1 = stimme überhaupt nicht zu, 5 = stimme voll und ganz zu] |
| topic selection expectation | mediator | Based on what you have experienced or heard, how confident are you that the journalist ...  [Gestützt auf das, was Sie erlebt oder gehört haben, wie sicher sind Sie sich, dass der Journalist …] |  |
|  |  | ... will interview you on a topic for which you are scientifically recognized  [… Sie zu einem Thema befragen wird, für das Sie wissenschaftlich ausgewiesen sind] | 1 = not confident at all, 5 = very confident  [1 = gar nicht sicher, 5 = sehr sicher] |
|  |  | … will give appropriate space to what you consider to be the most important aspects of the topic.  [… den aus Ihrer Sicht wichtigsten Aspekten der Thematik den angemessenen Raum einräumen wird] | 1 = not confident at all, 5 = very confident  [1 = gar nicht sicher, 5 = sehr sicher] |
|  |  | ...will contact you on a topic that is actually of general importance  […Sie zu einem Thema kontaktieren wird, das tatsächlich von allgemeiner Bedeutung ist] | 1 = not confident at all, 5 = very confident  [1 = gar nicht sicher, 5 = sehr sicher] |
|  |  | ...will contact you on a topic that is amenable to scientific analysis  […Sie zu einem Thema kontaktieren wird, das der wissenschaftlichen Analyse zugänglich ist] | 1 = not confident at all, 5 = very confident  [1 = gar nicht sicher, 5 = sehr sicher] |
| accuracy expectation | mediator | Based on what you have experienced or heard, how confident are you that the journalist ...  [Gestützt auf das, was Sie erlebt oder gehört haben, wie sicher sind Sie sich, dass der Journalist …] |  |
|  |  | ... will reproduce the findings you have reported factually correctly  [… die von Ihnen berichteten Erkenntnisse sachlich richtig wiedergeben wird] | 1 = not confident at all, 5 = very confident  [1 = gar nicht sicher, 5 = sehr sicher] |
|  |  | ... will not make any errors in the content of your input  [… keine inhaltlichen Fehler bei der Wiedergabe Ihrer Inputs machen wird] | 1 = not confident at all, 5 = very confident  [1 = gar nicht sicher, 5 = sehr sicher] |
|  |  | .... will not pass off any untrue claims as scientific evidence  […. keine unwahren Behauptungen als wissenschaftliche Evidenz ausgeben wird] | 1 = not confident at all, 5 = very confident  [1 = gar nicht sicher, 5 = sehr sicher] |
| fact selection expectation | mediator | Based on what you have experienced or heard, how confident are you that the journalist ...  [Gestützt auf das, was Sie erlebt oder gehört haben, wie sicher sind Sie sich, dass der Journalist …] |  |
|  |  | ... will concentrate on the scientific facts that are really important  [… sich auf die wissenschaftlichen Fakten konzentrieren wird, die wirklich wichtig sind]. | 1 = not confident at all, 5 = very confident  [1 = gar nicht sicher, 5 = sehr sicher] |
|  |  | ... will reproduce your input in appropriate completeness  [… Ihre Inputs in angemessener Vollständigkeit wiedergeben wird] | 1 = not confident at all, 5 = very confident  [1 = gar nicht sicher, 5 = sehr sicher] |
|  |  | ... will take up the state of research in the necessary breadth  [… den Forschungsstand in der notwendigen Breite aufgreifen wird] | 1 = not confident at all, 5 = very confident  [1 = gar nicht sicher, 5 = sehr sicher] |
|  |  | ... will not treat the topic one-dimensionally  [… das Thema nicht eindimensional behandeln wird] | 1 = not confident at all, 5 = very confident  [1 = gar nicht sicher, 5 = sehr sicher] |
| appropriate evaluation expectation | mediator | Based on what you have experienced or heard, how confident are you that the journalist ...  [Gestützt auf das, was Sie erlebt oder gehört haben, wie sicher sind Sie sich, dass der Journalist …] |  |
|  |  | ... will plausibly justify his/her own judgement on the state of research  [… sein eigenes Urteil über den Stand der Forschung plausibel begründen wird] | 1 = not confident at all, 5 = very confident  [1 = gar nicht sicher, 5 = sehr sicher] |
|  |  | ... will correctly explain the relevance of scientific expertise to the topic to his/her audience  [… seinem Publikum die Relevanz wissenschaftlicher Expertise für das Thema richtig erläutern wird] | 1 = not confident at all, 5 = very confident  [1 = gar nicht sicher, 5 = sehr sicher] |
|  |  | ... will correctly assess the scientific value of your expertise on the topic  [… den wissenschaftlichen Stellenwert Ihrer Expertise zum Thema korrekt beurteilen wird] | 1 = not confident at all, 5 = very confident  [1 = gar nicht sicher, 5 = sehr sicher] |
| impact expectation | mediator | Based on what you have experienced or heard, how confident are you that the journalistic contribution ...  [Gestützt auf das, was Sie erlebt oder gehört haben, wie sicher sind Sie sich, dass der journalistische Beitrag …] |  |
|  |  | ... will lead to a better understanding of the topic among the audience  [… zu einem besseren Verständnis der Thematik im Publikum führen wird] | 1 = not confident at all, 5 = very confident  [1 = gar nicht sicher, 5 = sehr sicher] |
|  |  | … will improve the audience's understanding of the scientific basis of the topic  [… das Verständnis für die wissenschaftlichen Grundlagen des Themas im Publikum verbessern wird] | 1 = not confident at all, 5 = very confident  [1 = gar nicht sicher, 5 = sehr sicher] |
|  |  | ... will enable the audience to participate constructively in discussions on the topic  [… es dem Publikum ermöglichen wird, sich konstruktiv an Diskussionen zum Thema zu beteiligen] | 1 = not confident at all, 5 = very confident  [1 = gar nicht sicher, 5 = sehr sicher] |
| Willingness | dependent variable | How willing are you currently as a scientist to talk to the news media?  [Was würden Sie sagen, wie groß ist derzeit Ihre Bereitschaft, sich als Wissenschaftlerin oder Wissenschaftler in journalistischen Massenmedien zu äußern?] | 1 = not at all willing, 5 = very willing  [1 = gar keine Bereitschaft, 5 = sehr große Bereitschaft] |
| Intention | dependent variable | I will express my views in the mass media when I have the opportunity to do so  [Ich werde mich in den journalistischen Massenmedien äußern, wenn ich die Möglichkeit dazu habe.] | 1 = very unlikely, 5 = very likely  [1 = sehr wahrscheinlich, 5 = sehr unwahrscheinlich] |

Table A2: Test of measurement invariance for CFA with correlated factors

| Model | Chi-Square | df | p | CFI | RMSEA | SRMR | ∆ X^2^ (∆ df) | ∆ CFI | ∆ RMSEA | ∆ SRMR |
| --- | --- | --- | --- | --- | --- | --- | --- | --- | --- | --- |
| M1  Configural ME | 2274.632 | 545 | ˂ .001 | .959 | .063 | .042 |  |  |  |  |
| M2  Metric ME | 2350.942 | 593 | ˂ .001 | .959 | .061 | .045 | 76,310 (48)*** | .000 | .002 | .003 |
| M3  Scalar ME | 2508.878 | 641 | ˂ .001 | .956 | .060 | .042 | 157,936 (48)*** | .003 | .001 | .003 |
| M4  Strict ME | 2883.362 | 709 | ˂ .001 | .949 | .062 | .045 | 374,484 (68)*** | .007 | .002 | .003 |
| Note: *** p ˂ .001 | |  |  |  |  |  |  |  |  |  |

Table A3: Test of measurement invariance for CFA with second order factor

| Model | Chi-Square | df | p | CFI | RMSEA | SRMR | ∆ X^2^ (∆ df) | ∆ CFI | ∆ RMSEA | ∆ SRMR |
| --- | --- | --- | --- | --- | --- | --- | --- | --- | --- | --- |
| M1  Configural ME | 2369.347 | 570 | ˂ .001 | .958 | .063 | .044 |  |  |  |  |
| M2  Metric ME | 2497.001 | 634 | ˂ .001 | .956 | .061 | .053 | 127.654 (64)*** | -.002 | -.002 | .009 |
| M3  Scalar ME | 2653.975 | 678 | ˂ .001 | .953 | .060 | .051 | 156.974 (44)*** | -.003 | -.001 | .002 |
| M4  Strict ME | 3027.392 | 746 | ˂ .001 | .946 | .062 | .053 | 373.417 (68)*** | .002 | .002 | .002 |
| Note: *** p ˂ .001 | |  |  |  |  |  |  |  |  |  |
